# Supplementary material for: The effect of therapeutic potential and safety of bone marrow-derived against adipose-derived mesenchymal stem cells in aged mice associated with septic arthritis
Source: PLoS One. 2025 Nov 26;20(11):e0335011. doi: 10.1371/journal.pone.0335011 (PMC12654889; doi:10.1371/journal.pone.0335011)
Supplement: Attachment — Submitted filename: Response to Reviewer 2.docx [file pone.0335011.s003.docx]

Response to Reviewer 2 (Report 2)

The authors aimed to address a significant and relevant biological question and have employed robust analytical techniques; however, there are notable areas that require improvement. The manuscript, in its current form, presents significant methodological and reporting deficiencies that must be addressed. Without clear experimental design details, proper stem cell characterization, statistical clarity, and complete data presentation, the study’s conclusions remain unsubstantiated. I recommend major revisions to improve the manuscript’s scientific validity before it can be reconsidered for publication. The study has potential relevance but is not presented or documented properly, it’s crucial to address the bare minimum requirements to improve its quality and credibility.

**Comments 1:** Clearly state the methodology and ensure that all treatment groups are properly defined. Revise for clarity and completeness.

**Response 1**: The methodology has been revised for clarity and all treatment groups have been properly defined. Please refer to the revised file.

**Comments 2:** Ensure that all abbreviations are defined upon first use in the abstract and text (e.g., ADMSC and BMSC).

**Response 2:** All abbreviations have been defined as suggested.

**Comments 3:** Please indicate the generation of MSCs used for the experiment. In addition, please provide a morphologic map of the MSCs and a file in FCS format for flow cytometry.

**Response 3:** The generation has been provided as suggested (page 6) and we have also provided morphologic map of the MSCs, please refer to pages 14-15. The file in FCS format for flow cytometry has been provided as supplementary file.

**Comments 4:** Line 54: Verify the reference that claims BM-MSCs as the gold standard.

**Response 4:** The reference has been provided and verified as suggested. The supporting reference has been added. Please refer to line 57-58 in the revised file.

**Comments 5:** Line 68: Add a supporting reference.

**Response 5:** The supporting reference has been added. Please refer to line 72-73 in the revised version.

**Comments 6:** Specify the total number of animals used, including their sex and distribution across groups. Clearly mention how many animals were used for stem cell isolation.

**Response 6:** Number of animals used including gender have been specified (page 5 line 107-122 and line 171-174).

**Comments 7: T**he study lacks characterization of stem cell markers—explain why specific markers for ADMSC and BM-MSC were not used.

**Response 7:** The information on characterization of stem cell markers have been provided. Please refer to page 7 and pages 12-13.

**Comments 8:** Minimum reporting criteria for stem cell characterization have not been met. Include data to support MSC characterization

**Response 8:** The data to support MSC characterization has been included as suggested. Please refer to page 7 and pages 12-13.

**Comments 9: T**he current description lacks clarity. Introduce a separate heading for Experimental Design.

**Response 9:** A separate heading for Experimental Design has been introduced as suggested.

**Comments 10:** Clearly define ADMSC1, ADMSC2, BMMSC1, and BMMSC2, explaining their roles in the study.

**Response 10:** ADMSC1, ADMSC2, BMMSC1, and BMMSC2 cells have been clearly defined as suggested.

**Comments 11:** Line 127: Add a reference for the dose of stem cells used.

Line 164: Specify the statistical methods used, including the software, significance level, and confidence interval.

**Response 11:** References used have been added. Please refer to page 9. The statistical methods used, including the software, significance level, and confidence interval have been provided on pages 12-13.

**Comments 12:** Mention the primers used or provide a reference for RNA quantification methods.

**Response 12:** The primers used are provided on page 11.

**Comments 13: T**he ISCT criteria for MSC characterization have not been fully met—include additional characterization data to confirm MSC identity and differentiate ADMSC from BM-MSC.

**Response 13:** We have provided more details and results in fulfilment of ISCT criteria for MSC characterization. Please refer to page 7-8.

**Comments 14:** Clearly mention the histological scoring method used.

**Response 14:** We have provided the histological scoring method used on page 13.

**Comments 15:** Include necessary references for materials and methods.

**Response 15:** All necessary references have been included.

**Comments 14:** Histopathology Figures:

o Ensure scale bars and magnifications are specified.

o Specify the organ being analyzed in the figure legends.

**Response 14:** Histopathology figures have been improved as suggested.

**Comments 15:** Sox9 Expression: The non-specific binding observed in BM-MSC groups needs to be clarified. Provide an alternative figure or additional data for validation.

**Response 15:** Alternative figure has been provided as suggested. Please refer to pages 32-33.

**Comments 16:** • Fold Change Calculations:

o Specify the method used for fold change calculations.

o Include details on statistical analysis of fold changes.

**Response 16:** The method used has been included as requested.

**Comments 17:** Pain & Inflammation Data:

o The manuscript mentions measurements in Materials & Methods, but results are not provided.

o If included in supplementary material, mention this in the main text and provide relevant details.

**Response 17:** Please refer to page 18-19 and figure **6.**

**Comments 18:** Figures 3, 4, and 7: Mark statistical significance on graphs.

**Response 18:** The statistical significances have been marked on graphs as requested.

**Comments 19:** Using only one pro-inflammatory marker is insufficient to draw reliable conclusions. Consider adding more markers to strengthen the findings.

**Response 19:** Tumor necrosis factor-alpha (TNF-a) and matrix metalloproteinase-13 (MMP-13) pro-inflammatory markers used in this study.

**Comments 20:** Give data of all scores used

• Give data of real time RNA quantification results -CT values

**Response 20:** The real time RNA quantification results -CT values have been provided. Please refer to page 28 and fig 8.

Thank and appreciate time and effort that the reviewer put to improve our work.
