# Supplementary material for: The effect of therapeutic potential and safety of bone marrow-derived against adipose-derived mesenchymal stem cells in aged mice associated with septic arthritis
Source: PLoS One. 2025 Nov 26;20(11):e0335011. doi: 10.1371/journal.pone.0335011 (PMC12654889; doi:10.1371/journal.pone.0335011)
Supplement: Attachment — Submitted filename: Response to Reviewer_October 2025.docx [file pone.0335011.s005.docx]

**Response to Reviewer**

**Comments 1:** Lines 111–112: The text states that only the treated groups were replicated. Please clarify what this means. Were the untreated and control groups not replicated? If so, why? Replication should ideally be consistent across all groups to avoid bias.

**Response 1**: The statement has been clarified. This is because each group was replicated four times. Please, refer to line 112-114 in the revised file.

**Comments 2:**  Line 120: It is stated that the untreated groups received no therapy. However, if all four untreated subgroups (Un-BM1, Un-BM2, Un-AD1, Un-AD2) only received PBS, the distinction between them is unclear. Why are they separately labeled as Un-BM1, Un-BM2, etc.? Please justify the rationale.

**Response 2**: In line 110-112, Un-BM1 and Un-BM2 represent untreated bone marrow-derived MSCs (type 1 and 2, defined in the next paragraph), while Un-AD1 and Un-AD2 represent untreated adipose-derived MSCs (type 1 and 2). The only difference is that the control group received PBS without arthritis induction, while the untreated groups received PBS after arthritis induction. Please refer to lines 126-128.

**Comments 3:** Line 125: Based on the description, the only difference is that the control group received PBS without arthritis induction, while the untreated groups received PBS after arthritis induction. If correct, please state this explicitly to avoid confusion.

**Response 3**: Yes, that is correct. This clarification has been added to the revised file. We appreciate it.

**Comments 4:** Line 172: Please clarify whether separate donor mice were used for MSC isolation or whether experimental mice themselves were used as donors. If separate donors were used, how many animals were involved? This information is important for ethical and methodological transparency.

**Response 4**: A separate mice were used as donor. The detail information regarding this has been added (lines 76-89). Please refer to revised file.

**Comments 5:** Line 219: The manuscript mentions differentiation but does not provide details of the differentiation assay. Please describe the protocols followed for osteogenic, chondrogenic, and adipogenic differentiation, including induction media and culture duration.

**Response 5**: The detail protocols have been provided in the revised file in lines 242-277.

**Comments 6:** Lines 219–220: Only Alizarin Red staining for osteogenesis is mentioned. What staining protocols were used for adipogenesis (e.g., Oil Red O) and chondrogenesis (e.g., Alcian Blue or Safranin O)? Please include full details.

**Response 6**: The detail protocols have been provided in lines 242-277.

**Comments 7:** Line 222 (Figure 1): Figure 1 is referred to in the text but does not appear in the manuscript. Kindly ensure that all referenced figures are included.

**Response 7**: Something must have happened in the system but attached all figures at submission. However, we have re-attached Figure 1 in this revision.

**Comments 8:** Line 245: The manuscript states that arthritis was induced and MSCs were injected within 24 hours. How was arthritis confirmed to have developed before treatment administration? Were clinical, histological, or imaging assessments used, or was this timing based on prior references? Please clarify.

**Response 8**: We have clarified and provide detail how we confirmed the development of arthritis before treatment administration. Please, refer to 352-364.

**Comments 9:** Lines 248–250: The description of untreated groups remains confusing here. Please clarify exactly what was administered and why these subgroups differ in labeling.

**Response 9**: The statement has been revised accordingly for more clarity. Please refer to lines 347-349.

**Comments 10:** Lines 260–261: Please provide details of the protocols for Hematoxylin & Eosin, Safranin O, Masson’s Trichrome, COL2A1, and SOX9 staining/ IHC, including duration, concentrations, and source references.

**Response 10**: The detail protocols have been provided in lines 280-324. Please refer to page 12-14.

**Comments 11:** Line 454: The phrase “untreated cell” is unclear. Does this mean cells were administered but not pre-treated, or does it refer to groups that received no MSCs at all? Kindly clarify terminology.

**Response 11**: The statement has been revised by removing the confusing phrase. Please refer to line 564.

**Comments 12:** Line 687: The phrase should be revised to “used to” for grammatical accuracy.

**Response 12**: It has been revised as suggested.

**Comments 13:** Line 874: Please ensure that “BM-MSC” is abbreviated consistently at its first mention and uniformly used throughout the manuscript.

**Response 13**: It has been revised accordingly.

We thank the reviewer for time and effort in helping us improve our work.
